# The risk of cumulative radiation exposure in chest imaging and the advantage of bedside ultrasound

**DOI:** 10.1186/s13089-015-0020-x

**Published:** 2015-03-28

**Authors:** Luna Gargani, Eugenio Picano

**Affiliations:** Institute of clinical Physiology, National Research Council, via Moruzzi 1, 56124 Pisa, Italy

**Keywords:** Radiation exposure, Chest imaging, Point of care, Lung ultrasound

## Abstract

The increasing use and complexity of imaging techniques have not been matched by increasing awareness and knowledge by prescribers and practitioners. Imaging examinations that expose to ionizing radiation provide immense benefits when appropriate, yet they may result in an increased incidence of radiation-induced cancer in the long-term. The radiation issue is relevant not only for the individual patient but also for the community because small individual risks multiplied by millions of examinations become a significant population risk. As recently highlighted by recent European and American Guidelines, the long-term risk associated with radiation exposure should be considered in the risk-benefit assessment behind appropriate prescription of diagnostic testing.

## Introduction

The fast and strong development of medical imaging represents an exceptional success in the history of medicine. Imaging tests are now the mainstay of our diagnostic approach in many diseases: they allow detection of anatomical and physiological abnormalities often unmasking clinically silent conditions, they are crucial to guide treatment and they can be life-saving. Nevertheless, the widespread use of imaging techniques poses some relevant concerns about the unsustainable society costs and the non-negligible health risks of an inappropriate use [1-3].

## Review

### Why is radiation an issue?

Medical radiation from X-rays and nuclear medicine is the largest man-made source of radiation exposure in Western countries [4]. Ionizing radiation including X-rays and *γ*-rays are well-known proven carcinogens, according to the classification of the World Health Organization’s International Agency for Research on Cancer [5]. Radiation’s deleterious effects are typically classified as *stochastic effects* that are due to radiation-induced mutations and *deterministic effects* (i.e. tissue reactions) due to radiation-induced cell death. Deterministic effects only occur above a threshold level of radiation, which is generally higher than levels occurring from a single non-invasive imaging procedure. The risk of developing cancer deriving from radiation exposure is mainly stochastic, which means that it may occur without a specific threshold level, although the magnitude of this risk remains unclear, particularly at very low doses. The risk is also cumulative.

According to the risk estimates released in the Seventh Report of the Committee to Assess Health Risks from Exposure to Low Levels of Ionizing Radiation, the attributable risk of cancer is 1/750 for 15 mSv exposure [4]. Radiation-induced cancers typically do not occur until one or two decades or even longer after exposure. Thus, any increase in cancer occurrence due to medical imaging may not be expected to be evident for many years after exposures. Radiological dose estimate can be expressed as multiples of a single postero-anterior chest X-ray (equal to 0.02 milliSievert, mSv), as originally suggested by the UK College of Radiologists and endorsed in the European Commission referral guidelines on medical imaging [6] and - more recently - by the European Society of Cardiology position paper on medical radiation [2] (Table 1).

**Table 1:** **Standard average estimated radiation doses of common non-invasive chest imaging in adults (modified from refs.** [2] **and** [21]**)**

**Diagnostic procedures**	**Effective dose (mSv)**	**Equivalent CXRs number**
Radiology		
Chest radiography (single postero-anterior film)	0.02	1
Chest CT	6 to 8	300 to 400
64-slice coronary CT	15 (3 to 32)	750 (150 to 1,600)
Calcium score	3 (1–12)	150
Nuclear medicine		
^99m^Tc-Sestamibi (1100 MBq, 1 day) stress-rest	9.4	470
^201^Thallium stress/rest reinj. (185 MBq, double injection)	40.7	2,035
PET N-13 ammonia stress-rest (1100 MBq)	2.4	120
PET F-18 FDG rest (400 MBq, viability)	8	400
^133^Xenon (400 MBq, lung ventilation)	0.4	20
^99m^Tc-MAA (185 MBq, lung perfusion)	2	100

#### Women and children first

For each dose, the risk varies greatly depending on the age (lower in the elderly) and gender (about 38% higher in women than in men at all ages of life) [2]. Children are at substantially higher risk than adults because they have more rapidly dividing cells and a greater life expectancy. Thus, an infant or child patient has a longer lifetime risk for developing radiation-induced cancers than adult patients. A recent study has reported that the average child in the US receives seven medical imaging tests involving radiation by the time he or she reaches the age of 18 [7].

The issue of radiological responsibility in children was recently addressed in the US with the *Image Gently*, *Step Lightly* Campaign, focused on the risks of unnecessary and excessive medical radiation exposure from interventional radiology administered to paediatric patients [8]. The European directive 2013/59/euratom (art. 61) underlines that special attention shall be given to quality assurance programmes and the assessment of dose in medical exposure of children [9].

#### How much are we aware?

Many data clearly show that both patients and doctors - including radiologists, cardiologists, paediatricians, and nuclear cardiologists - have until recently been largely unaware of the long-term risk of the imaging studies they commonly use [10,11]. Patients obviously have the right to know, according to medical deontological code and the law [9], but our informed consent forms are usually reticent or impossible to understand in their parts addressing radiological risk [12,13]. The White Paper on Radiation Dose in Medicine underlines that ‘It is incumbent on radiologists to assume the responsibility for their patient’s safety with regard to radiation exposure. They should also educate their patients on these issues so they may make informed decisions about their health care’ [14]. The White Paper states also that ‘Although some referring physicians are very knowledgeable regarding safety issues and incorporate such information into their imaging decisions, others have had little or no training in radiation exposure and do not routinely consider this factor when ordering imaging examinations’ [14]. Even though the law already imposes specific behaviours designed to protect against the dangers arising from exposure to ionizing radiation [9], some wrong practices are still very common (Table 2).

**Table 2:** **Radiation in paediatric imaging: current and future approaches (modified from ref.** [23]**)**

	**Current approach**	**What we need**
Patient		
Culture	More (exams) is better	Less (dose) is better
Radiation history	Absent	Present
Radiological informed consent	Absent	Present and informative
Received dose in report	Missing	Mandatory
Organ dose	Ignored	Considered
Doctor/scientist		
Optimizing dose	Matter of investigation for physicists	Preventing cancer
Technology upgrading	Focused on short-term costs	Focused on long-term risks
Radiological risk estimation	Population-based	Personalized
Dose reading	Offline, months later	Online, real time

### Chest imaging

The number of CT scans of all types performed in the USA has quadrupled since 1993, and the same increasing trend has also been observed in Europe [15]. The increase in imaging utilization has led to a nearly sixfold increase in the per capita dose of radiation from medical imaging occurred in the USA between 1982 and 2007. Chest imaging exposing to ionizing radiation includes chest X-ray and chest CT but also coronary CT angiography, nuclear cardiology, invasive coronary angiography and angioplasty, which account for a significant percentage of all diagnostic examinations. The chest is the most frequently evaluated region of the body in children [15]. CT scans account for 8% of exams in the paediatric population, and data show that they are often performed without adjusting exposure parameters to weight, resulting in up to 50% of the dose being unnecessary [15]. Moreover, radiological chest imaging should be especially well justified and carefully optimized in women because from the radiobiological viewpoint the female breast is a highly radiosensitive organ [4].

The radioprotection issue is intrinsically linked also to the appropriateness issue. Some recent studies have shown significant percentages of partially or totally inappropriate radiological examinations, even in top-level academic medical centres [16,17]. Exams that are not appropriate should not be performed, but exams that are not appropriate and yield even a very low biological risk to the patient should be avoided. The inappropriateness of imaging techniques, especially when exposing to ionizing radiation, is becoming economically and socially unsustainable.

Bedside point-of-care ultrasonography is emerging as a crucial bedside tool that allows timely decision-making, especially in critically ill patients. Besides conventional ultrasonography, point-of-care echo is a new diagnostic approach to help the attending physicians answer specific questions that are clinically focused and need rapid response.

This new way of using the probe as an extension of the examining hand and ear is gaining consensus especially in intensive care units and emergency departments but also for the management of more stable patients. CT definitively remains the gold standard imaging tool for most conditions, but the implications of transferring a critically ill patient out of ICU have stimulated the scientific community and physicians to approve this innovative and revolutionary approach to ultrasound [18]. Accordingly, the recent introduction of lung ultrasound in the clinical practice has been paradigmatic, and from a counterintuitive use of sonography, the technique is now being used more and more frequently, both in acute and chronic settings [19]. When we choose lung ultrasound as a first-line exam instead of more complex and costly chest CT, it is not for radioprotection issues, but for the advantages of a bedside, rapidly available, highly versatile technique. However, it may be also useful to reduce our patients’ exposure and, in specific populations, it may be the test of choice for the initial screening of interstitial lung disease [20]. In patients with chronic conditions, an extensive use of CT for a tight follow-up is indeed limited by the non-negligible cumulative radiation exposure of this examination.

It is crucial that the new generations of physicians and health care providers are no longer unaware of the basics of radioprotection. The White Paper itself suggests a profound remodelling of radioprotection teaching. It is advised that ‘education of future referring physicians on radiation exposure to patient during diagnostic imaging should begin during medical school. The goal is to develop the awareness of radiation exposure in students during training. Any clinician should be steered toward an imaging regimen that minimizes radiation.’ [14] The two very recent documents of the American Heart Association and European Society of cardiology both witness the attention paid by non-radiological societies to the radiation issue and emphasize concordantly that a responsible use of medical radiation should rest on three pillars: Justification (only appropriate tests should be done), Optimization (only the right dose should be used) and Education (both doctors and patients should be aware of what they do, in terms of radiological doses and risks) [2,3]. In chest imaging, this means very pragmatically to implement, whenever possible, the expertise to shift the diagnosis from ionizing imaging testing to non-ionizing techniques, for instance with lung ultrasound integrating - and in selected case replacing - chest X-ray and chest CT whenever possible [21].

## Conclusions

Underlining the importance of radiological awareness does not translate in an underestimation of the crucial, irreplaceable role of some imaging techniques and examinations that can be life-saving and have dramatically changed the management of some serious illness. The risks of a test should always be weighed against the risks if a disease remains undetected, detected at a later stage, incorrectly prognosticated or suboptimally treated [22]. It is however not recommended to perform tests involving ionizing radiation when the desired information can be obtained with a non-ionizing test with comparable accuracy [2]. We cannot only rely on the benefit of the medical procedures we propose to our patients, but we have to include long-term cancer risks in the risk-benefit assessment of diagnostic or therapeutic testing. The cultural benefit of remodelling our awareness about medical radiation exposure will be immense to enhance the protection of patients and physicians themselves and to ultimately provide better patient care. As stated in the recent European Society of Cardiology Position paper on use of medical radiation in cardiovascular imaging, a smart physician ‘cannot be afraid of the essential and often life-saving use of medical radiation, but must be very afraid of radiation unawareness’ [2].
